# Identification of A Novel Mutation of SHORT Syndrome: A Case Report

**DOI:** 10.1002/ccr3.70820

**Published:** 2025-08-25

**Authors:** Quynh Thi Vu Huynh, Tuong Trong Luong, Ho Tran Ban, Van Tran

**Affiliations:** ^1^ Department of Pediatrics University of Medicine and Pharmacy Ho Chi Minh City Vietnam; ^2^ Nephrology and Endocrinology Department Children's Hospital 2 Ho Chi Minh City Vietnam; ^3^ Department of Pediatric Surgery University of Medicine and Pharmacy Ho Chi Minh City Vietnam; ^4^ General Surgery Department Children's Hospital 2 Ho Chi Minh City Vietnam; ^5^ Medical Genetics Institute Vietnam

**Keywords:** endocrinology and metabolic disorders, genetics and genomics, healthcare management, pediatrics and adolescent medicine

## Abstract

SHORT syndrome is a rare inherited disease with 34 identified pathogenic or likely pathogenic *PIK3R1* mutations. The genotype–phenotype relationship remains inconsistent. Our case presents the first novel duplication that affects up to 25 nucleotides and truncates the PI3K protein, contributing valuable data to genetic understanding and characterization worldwide.

## 1 Introduction

SHORT syndrome is a rare genetic disorder inherited in an autosomal dominant pattern. The acronym “SHORT” highlights the typical features of the condition: S for short stature, H for hyperextensibility, O for ocular depression, R for Rieger anomaly, and T for delayed tooth development [1]. These characteristics were also prominent in the first cases reported by Gorlin in 1975 [2]. In addition, other common features of SHORT syndrome include characteristic facial appearance, intrauterine growth restriction, short stature, sensorineural hearing loss, and insulin resistance [3]. A significant proportion of SHORT syndrome cases occurred in individuals without a family history [4], suggesting a relatively high incidence of de novo mutations.

SHORT syndrome is caused by mutations in the phosphoinositide‐3‐kinase regulatory subunit 1 (*PIK3R1*) gene, which is located on the long arm of chromosome 5. The *PIK3R1* gene encodes the regulatory part of the enzyme phosphatidylinositol 3‐kinase (PI3K), which is mainly the p85α isoform [5]. Its catalytic component is the p110 kD subunit [6]. PI3K is an important enzyme in various cell growth signaling pathways, with one of its main functions being the PI3K/Akt signaling pathway. The interaction between insulin—IRS (insulin receptor substrate) complexes and p85α‐p110α molecules leads to the increased synthesis of PIP_3_ (phosphatidylinositol‐3,4,5‐trisphosphate) [7], which in turn induces the release of various enzymes essential for intracellular signaling pathways [8]. In SHORT syndrome, mutations in *PIK3R1* impair the inhibitory function of p85α, disrupting the interaction between this subunit and the IRS molecule [9] and leading to the impairment of the PI3K‐Akt signaling pathway.

The *PIK3R1* gene is located on the long branch of chromosome 5, at position 5q13.1 [1], and comprises a total of 16 exons [10]. According to the ClinVar database of the US National Institutes of Health, 34 mutations in the *PIK3R1* gene have been identified as pathogenic and likely pathogenic for SHORT syndrome. The majority of these are frameshift mutations (11/34). Other recorded mutations include splice site mutations (10/34), nonsense mutations (6/34), missense mutations (6/34), and in‐frame deletion (1/34) [11]. Regarding duplications causing SHORT syndrome, all duplications documented in the ClinVar database involve only one nucleotide [11].

Diabetes mellitus in SHORT syndrome is classified as lipoatrophic diabetes, a variant of type 2 diabetes resulting from genetic defects in insulin activity. To date, there are still no recommendations for the selection of medications to treat diabetes in SHORT syndrome. Previous reports suggested that insulin and metformin might be effective [12, 13, 14]. Additionally, SGLT2i (sodium‐glucose cotransporter‐2 inhibitor), sulfonylurea, and thiazolidinedione were also selected [12, 13, 14, 15, 16].

We reported a 12‐year‐old girl admitted for diabetes mellitus with typical manifestations of SHORT syndrome. Diagnosis was confirmed by the identification of a novel duplication in *PIK3R1*, affecting a chain of 25 nucleotides.

## 2 Case History/Examination

**FIGURE 1:**
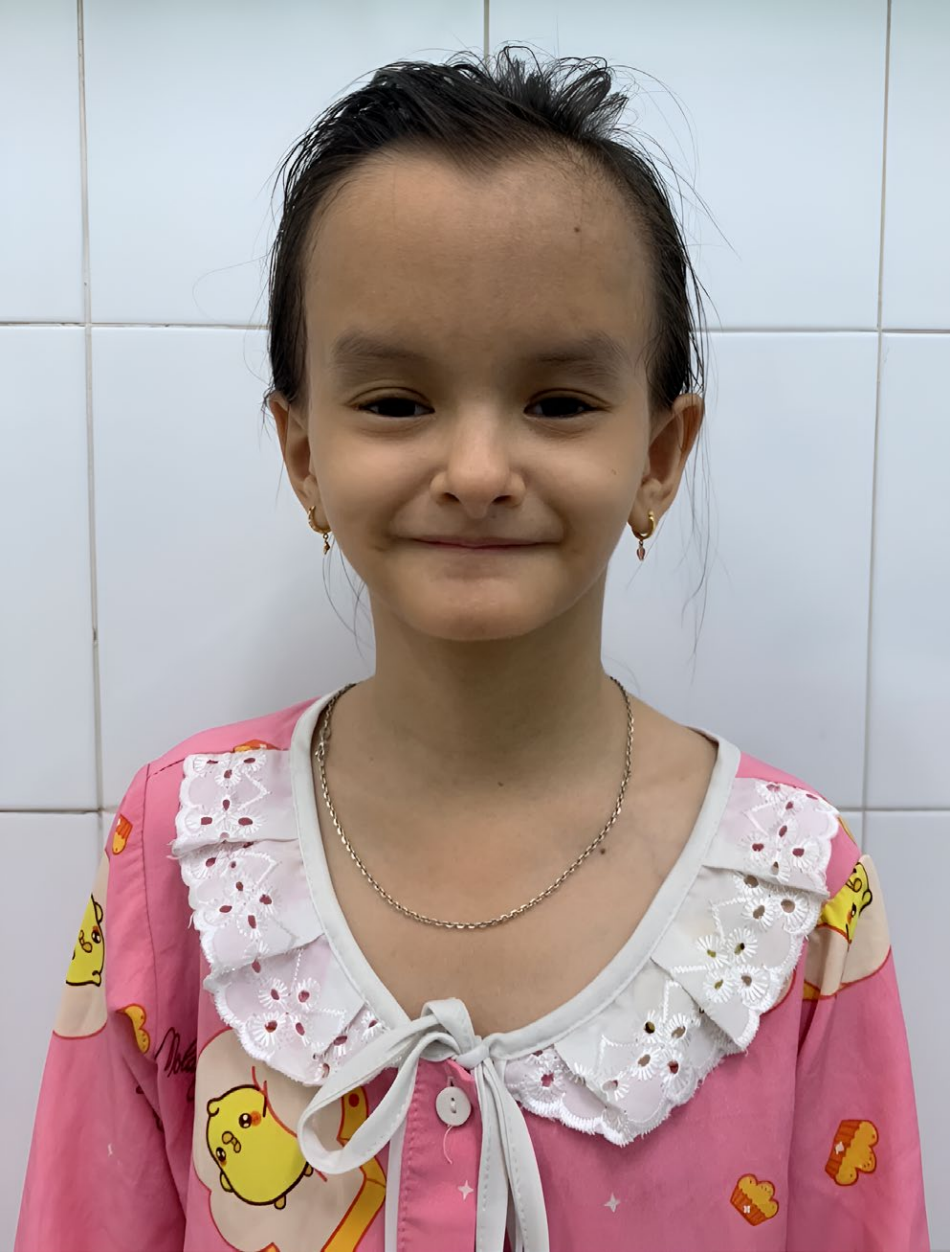
The patient's facial characteristics revealed dysmorphic features, including a broad forehead, ocular depression, prominent ears, and a downturned mouth, which are the typical components of the facial gestalt seen in SHORT syndrome.

The 12‐year‐old female patient was admitted to Children's Hospital 2 with symptoms of fatigue, loss of 2 kg in 3 weeks, polydipsia, and polyuria. Initial observation revealed the characteristic facial dysmorphism of SHORT syndrome with a broad forehead, ocular depression, prominent ears, and a downturned mouth, as shown in Figure 1.

The medical history revealed intrauterine growth retardation at 7 months of gestation and the cesarean section at 39 gestational weeks due to oligohydramnios. Her birth weight was 1,600 g. Her dental eruption was notably delayed, commencing at 24 months, with teeth exhibiting significant fragility and premature loss. The two younger siblings were healthy and did not have similar phenotypes.

## 3 Methods

### 3.1 Differential Diagnosis

Type 1 and type 2 diabetes are the two most common etiologies of diabetes mellitus. In this patient, type 1 diabetes was initially suspected due to pronounced symptoms, including weight loss, polydipsia, and polyuria, along with severe wasting malnutrition. The absence of acanthosis nigricans, a common indicator of insulin resistance, also supported type 1 diabetes. However, the coexistence of facial dysmorphism, joint hyperextensibility, and delayed dental eruption could not be explained by this etiology, prompting further evaluations for less common causes.

### 3.2 Investigations

During the examination, hyperextensibility was detected at two elbows. The patients exhibited signs of malnutrition with a weight of 21 kg (WA: −5.37 SD), height of 130 cm (HA: −3.55 SD), and BMI for age of −4.07 SD. Pubertal development was appropriate for the patient's age, with Tanner stage III for breasts, armpit hair, and pubic hair. Her initial random glycemia was elevated at 14.7 mmol/L. A diagnosis of diabetes was subsequently confirmed with an HbA1c level of 16.49%. The diabetes panel revealed a normal C‐peptide level (1.27 ng/mL) and negative diabetes‐related autoantibodies, which are not consistent with type 1 diabetes.

Due to the presence of severe short stature, a bone profile was obtained, which yielded normal results (Table 1). Bone age assessment was appropriate for the patient's chronological age. Screening for diabetes complications, including urinalysis, echocardiography, and an eye examination, yielded normal results, except for a slightly elevated urine albumin to creatinine ratio of 45.14 mg/g. The lipid panel was not obtained.

**TABLE 1:** Results of routine laboratory evaluations.

Serum biochemical parameter	Result	Reference range
25(OH)vitamin D (nmol/L)	78.57	50–150
Total calcium (mmol/L)	2.53	2.3–2.6
Phosphorus (mmol/L)	1.44	1.3–1.9
PTH (ng/L)	31	10–65
ALP (U/L)	262	141–460
Protein (g/L)	86	65–81
Albumin (g/L)	49	41–48
Sodium (mmol/L)	141	136–143
Potassium (mmol/L)	3.7	3.5–5.1
Urea (mmol/L)	5.1	2.6–6.8
Creatinine (μmol/L)	46	39.8–71.6

Abbreviations: ALP, alkaline phosphatase; PTH, parathyroid hormone.

Due to the presence of distinct facial dysmorphism at initial examination, a congenital etiology was suspected. The combination of typical facial appearance, short stature, joint hyperextensibility, and dental abnormalities further supported the clinical suspicion of SHORT syndrome. Therefore, the next‐generation sequencing targeting the *PIK3R1* gene was conducted, which detected the heterozygous mutation of the *PIK3R1* gene: NM_181523.3: c.1948‐1972dup, at nucleotide position 68,296,302–68,296,303, involving an insertion of 25 nucleotides (GGAGAGCAGTAAACAGGGCTGCTAT). This variant resulted in the predicted amino acid change NP_852664.1: p.Ala658GlyfsTer4. The variant was further confirmed by Sanger sequencing, showing a comparable heterozygous mutation. Genetic testing was not performed on the patient's parents or siblings due to the absence of characteristic dysmorphic features.

**FIGURE 2:**
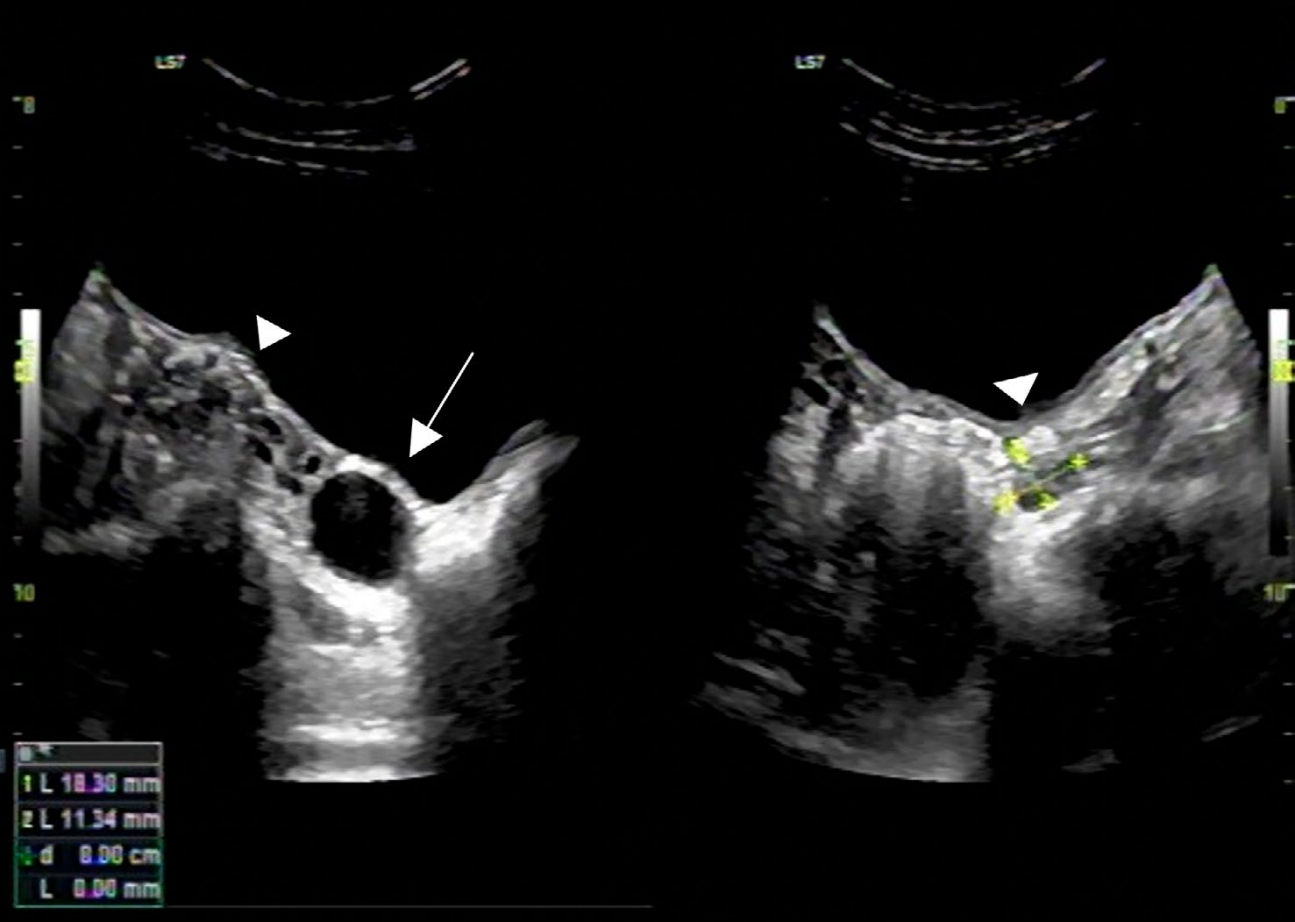
Ultrasound characteristics of a mass in the right ovary. A well‐circumscribed, anechoic cystic mass, measuring approximately 28 × 24 × 25 mm (solid arrow). The right ovary (arrowhead) measured 19 × 17 mm; the left ovary (arrowhead) measured 18 × 11 mm.

An otoacoustic emissions test was conducted to evaluate for hearing abnormalities associated with SHORT syndrome, which showed normal findings. Abdominal ultrasound detected a well‐circumscribed, anechoic cystic mass in the right ovary, measuring approximately 28 × 24 × 25 mm (Figure 2). The right ovary measured 19 × 17 mm; the left ovary measured 18 × 11 mm, and the uterine anteroposterior diameter was 8 mm. These findings were consistent with a functional ovarian cyst. Biomarkers, including α‐fetoprotein, lactate dehydrogenase, and β‐human chorionic gonadotropin, were not measured. As the patient was asymptomatic, a conservative approach with regular follow‐up was indicated. Currently, there is no clearly established association between ovarian cysts and SHORT syndrome.

### 3.3 Treatment

The patient was initially treated with a diabetic diet and a conventional regimen of subcutaneous short‐ and intermediate‐acting insulin, starting at a total dose of 0.5 units/kg/day.

## 4 Conclusions and Results

The insulin dose was gradually increased to 1.7 units/kg/day over two weeks. However, the glycemic control remained inadequate, with fasting plasma glucose ranging from 310 to 410 mg/dL. Metformin was subsequently prescribed at a dose of 1000 mg/day, resulting in the improvement of glycemic control, with fasting plasma glucose decreasing to a range of 250 to 270 mg/dL after three days.

## 5 Discussion

A study was conducted by Avila et al. in 2016 to evaluate the manifestations of SHORT syndrome in 32 patients. The findings revealed that all individuals exhibited similar characteristic facial features, including a triangular face, broad forehead, sunken eyes, prominent ears, low‐set ears, downward‐turned mouth, and micrognathia [3]. These traits were also fully manifested in our patient. However, Rieger anomaly and hyperextensibility were not consistent signs, with a prevalence of less than 50% [3, 4]. Our patient exhibited hyperextensibility; however, Rieger anomaly was not observed during eye examination.

In terms of genetics, the study of Avila et al. recorded nine different pathological mutations. The most notable mutation observed in 16 families, comprising 23 individuals, was c.1945C>T with the amino acid change from arginine to tryptophan at position 649 [3]. It was also the most common variant identified in the study of Thauvin–Robinet et al. [4], affecting five out of nine individuals.

**FIGURE 3:**
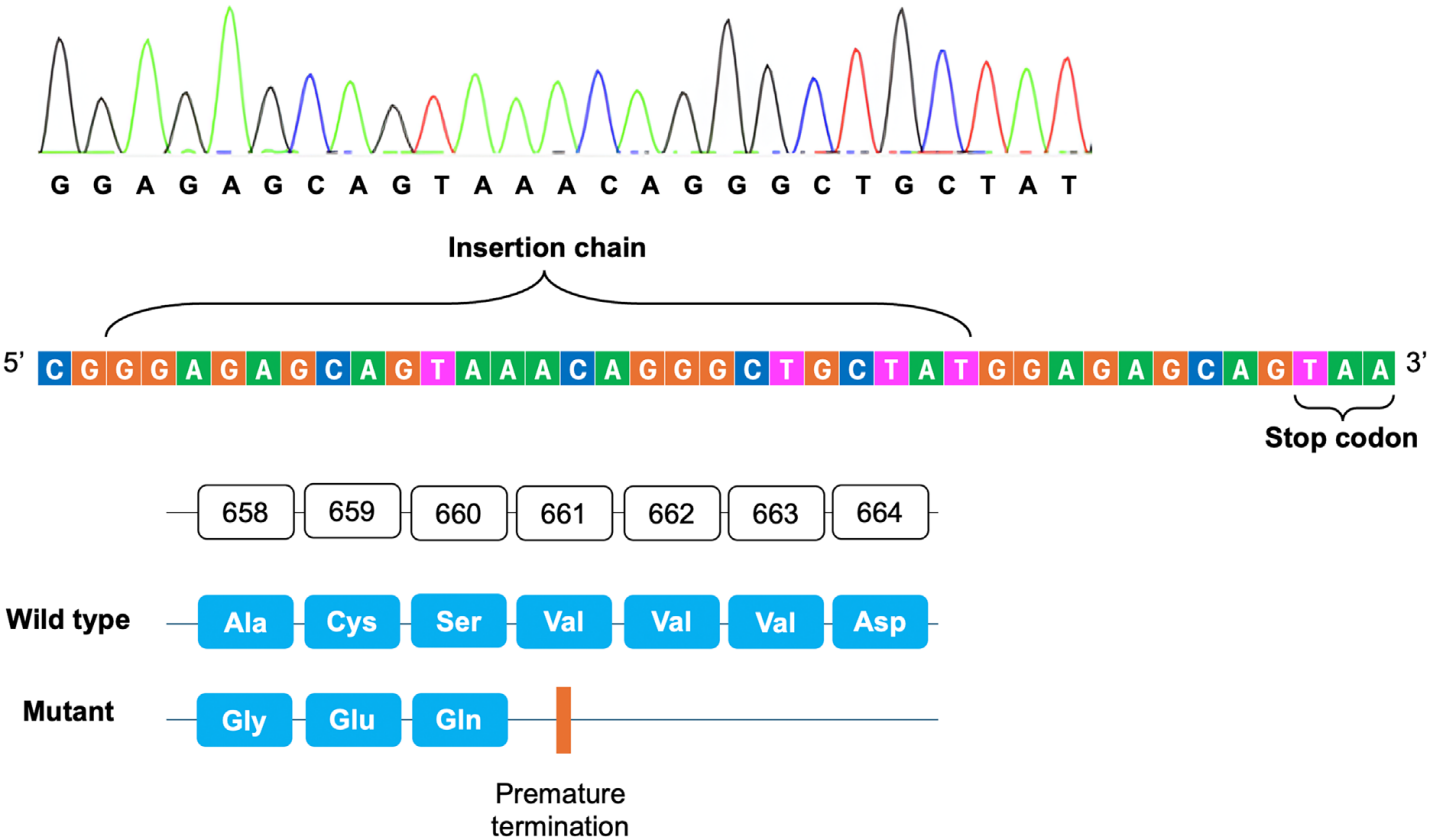
The comparison between the wild‐type and mutant PI3K proteins. In the mutant protein, alanine at position 658 is replaced with glycine, followed by the premature termination after two subsequent amino acids. As a result, the mutant protein is truncated compared to the wild‐type PI3K protein, which normally consists of 724 amino acids. The insertion chain was also confirmed by Sanger sequencing.

For our case, the identified variant is a frameshift mutation affecting exon 15 of the *PIK3R1* gene, which has not been previously reported in the ClinVar database. The variant is not found in the gnomAD genome [17] and it involves a loss‐of‐function mutation in the *PIK3R1* gene, which is a known mechanism for SHORT syndrome. These findings fulfill the PM2 and PVS1 criteria according to the guidelines for variant interpretation and classification of the American College of Medical Genetics (ACGM) [18]. Regarding molecular consequences, this mutation replaces alanine at position 658 with glycine and leads to a premature termination of translation, producing a truncated PI3K protein. A comparison between the wild‐type and mutant PI3K protein was illustrated in Figure 3, along with the location of the duplication chain in the DNA sequence.

According to the ClinVar database, there are currently four duplication mutations of the *PIK3R1* gene that are classified as pathogenic and likely pathogenic variants for SHORT syndrome according to the ACGM classification, with their specific details presented in Table 2.

**TABLE 2:** Four pathogenic and likely pathogenic duplications of SHORT syndrome in the ClinVar database.

Variant	Nucleotide change	Duplicated chain	Amino acid change	VCV accession number
1	NM_181523.3: c.1943dup	1 nucleotide	p.Arg649fs	VCV000060764.2
2	NM_181523.3: c.1710dup	1 nucleotide	p.Ile571fs	VCV000571336.6
3	NM_181523.3: c.1344dup	1 nucleotide	p.Leu449fs	VCV001393351.5
4	NM_181523.3: c.1708dup	1 nucleotide	p.Leu570fs	VCV003235185.1

Abbreviaiton: VCV, Variation ClinVar.

**FIGURE 4:**
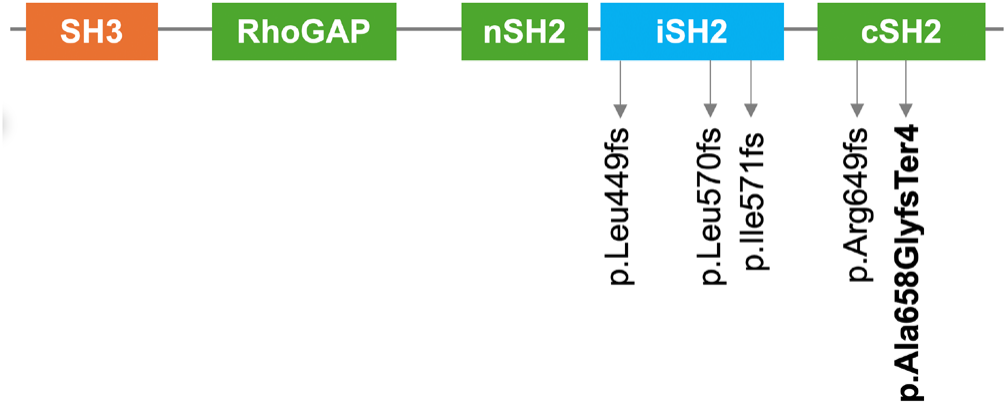
Schematic representation of PI3K protein structure highlighting the positions of pathogenic and likely pathogenic duplications in the ClinVar database, alongside the variant identified in our study. The PI3K protein contains five primary domains: Src homology 3 (SH3), RhoGAP, N‐terminal Src homology 2 (nSH2), inter Src homology 2 (iSH2) and C‐terminal Src homology 2 (cSH2).

Duplicated chains in all of these cases consist of only one nucleotide [11]. In our patient, the duplicated sequence spanned up to 25 nucleotides, making it the first recorded case of this length worldwide. A notable similarity among all documented duplications in SHORT syndrome was their consistent classification as frameshift mutations in terms of molecular consequences. Notably, variant 1 and our variant shared some common features, including the premature termination of PI3K protein and the closest proximity within protein domains compared with the other three duplications. These findings suggested some potential similarities in clinical manifestations between the two cases. Figure 4 illustrated the comparison of the location of our variant with the positions of the four mentioned duplications within PI3K protein domains.

Generally, the relationship between the genotype and phenotype of SHORT syndrome has not been clearly established. Among the four reported duplications, only the individual with variant 1 had clinical features documented in the study of Thauvin‐Robinet et al. [4] This patient, referred to as case 1, was a 60‐year‐old woman exhibiting short stature and sunken eyes, which comprise only two characteristics of the SHORT acronym. Rieger anomaly was absent; information regarding hyperextensibility and delayed tooth eruption was not provided. In contrast, our patient manifested four features of SHORT syndrome, compared to only two in case 1 [4]. However, typical facial characteristics and diabetes mellitus were common features in both cases. Due to the lack of clinical data for case 1, a comparison of the age of onset between the two cases was not possible. Regarding the c.1945C>T (Arg649Trp) mutation, all five individuals carrying this variant in the study of Chudasama et al. began exhibiting progeroid facial appearance specifically during infancy, accompanied by signs of partial lipodystrophy [9]. However, the genotype–phenotype association of this mutation was inconsistent in the study conducted by Avila et al. [3].

## Author Contributions

**Quynh Thi Vu Huynh:** conceptualization, methodology, supervision, writing – review and editing. **Tuong Trong Luong:** data curation, investigation, writing – original draft. **Ho Tran Ban:** investigation, methodology, resources. **Van Tran:** resources, validation.

## Consent

Written informed consent for participation in the study and for publication of photographs was obtained from the patient and the patient's guardian.

## Conflicts of Interest

The authors declare no conflicts of interest.

## Data Availability

The data that support the findings of this study are available from the corresponding author upon reasonable request.
